# Diversity analysis of oral and gut microbiota in osteoporotic rats

**DOI:** 10.1371/journal.pone.0320063

**Published:** 2025-06-03

**Authors:** Mingzhu Chen, Zihan Liao, Zhenjin Yang, Yanjie Li, Liya Ma, Jiangtian Hu

**Affiliations:** 1 Department of Orthodontics, Kunming Medical University School and Hospital of Stomatology, Kunming, Yunnan, China; 2 Yunnan Key Laboratory of Stomatology, Kunming, Yunnan, China; Hong Kong Baptist University, HONG KONG

## Abstract

The oral and gut microbiota had been shown to control bone metabolism and have a strong correlation with osteoporosis. However, to reveal the oral and gut bacteria characteristics in osteoporosis, further studies are still needed to investigate the relationship between oral and gut microbiota diversity and bone health in OVX-induced osteoporotic rats versus Sham-operated (Sham) rats. This study analyzed the oral and gut microbiota in OVX and Sham rats using 16S rRNA gene sequencing. We compared microbial diversity and composition between the two groups. There was an inverse association found between the number of bacterial taxa and bone mineral density (BMD) readings. The OVX group had considerably higher estimated diversity of both oral and gut microbiota than the Sham group. Firmicutes, Bacteroidota, Proteobacteria, and Actinobacteriota were the dominant phyla in both groups. The OVX group had a reduced ratio of Firmicutes in oral and gut microbiota compared to the Sham group (*p* <  0.05). OVX rats had a higher proportion of oral Bacteroidota but a lower proportion of gut microbiota. They also had a substantial drop in *Lactobacillus* in both oral and gut microflora (*p* <  0.01). The crosstalk between oral and gut microbiota may be important in the development of osteoporosis. Identifying novel biomarkers in the oral and gut microbiota could provide more information in osteoporosis and the intricate oral-gut-bone health interaction.

## Introduction

A common skeletal condition called osteoporosis (OP) is typified by decreased bone density and increased bone fragility. It primarily affects older adults, especially postmenopausal women, when a decrease in estrogen levels causes rapid bone loss [1,2]. According to studies, estrogen is essential for preserving bone health and density [3,4]. The risk of osteoporosis and fractures is increased when menopause results in estrogen insufficiency because it causes rapid bone loss and a marked increase in bone resorption [5,6]. OP has important consequences for oral health in addition to its direct effect on bone health [7,8]. Recent studies have demonstrated a reciprocal link between the oral microbiota and bone metabolism [9–11]. While the development of OP can alter the makeup and function of the oral microbiota, changes in the oral microbiome can also affect bone health [12,13]. Moreover, bone metabolism is significantly influenced by the gut microbiota [14,15]. While OP itself can alter the gut microbiota and affect systemic bone metabolism, dysbiosis in the gut microbiome may also play a role in the onset and progression of OP [16,17]. Systemic bone metabolism is impacted by the dynamic interaction between the gut and oral microbiomes, which includes the movement of microorganisms from the oral cavity to the gastrointestinal tract [17–19]. The purpose of this research is to examine how OP affects the gut and oral microbiomes and to clarify how these microbiomes interact and play a part in controlling bone metabolism. We want to shed light on possible biomarkers and treatment options for OP by investigating these relationships.

## Materials and methods

### Animals and experimental design

The current research runs from February to June 2024. The ethics committee of Kunming Medical University authorized the experimental animal protocol (approval number: kmmu20230702). All procedures followed the Institutional Animal Care and Use Committee’s (IACUC) recommendations. According to prior research, 8-week-old female Sprague-Dawley rats have achieved adulthood, with skeletal growth nearly complete and hormone levels regulated. As a result, they are often used in research on the pathophysiology of osteoporosis [20]. As a result, sixteen female Sprague-Dawley rats aged 8 weeks were obtained and housed in a pathogen-free (SPF) facility. After one week of acclimation, the sedated rats underwent either bilateral ovariectomy (OVX) or sham surgery (Sham) (n =  8 per group). Salivary and intestinal microbiota were collected 12 weeks after the operation and stored in liquid nitrogen. Rats were sacrificed via CO₂ inhalation and cervical dislocation, and samples were collected for further testing.

### Microcomputed tomography analysis

The rat femurs were preserved in 4% paraformaldehyde for 72 hours at 4°C before being imaged with the SkyScan 1176 scanner (Bruker, Karlsruhe, Germany). Data Viewer (version 1.5.6) and CTAn (version 1.18) were used to examine bone properties. Bone mineral density (BMD) was computed using two hydroxyapatite (Ca10(PO4)6(OH)2) models, yielding values of 0.250 g/cm³ and 0.750 g/cm³. The region of interest (ROI) within the femoral trabeculae was determined as a horizontal section beginning 1.00 mm from the distal growth plate and extending toward the proximal femur. Trabecular metrics, such as BMD, bone volume (BV), bone volume-to-total volume ratio (BV/TV), trabecular number (Tb.N), trabecular thickness (Tb.Th), and trabecular spacing (Tb.Sp), were measured and quantified. To ensure reproducibility, two separate researchers conducted all analyses.

### Histological analysis

The femur was decalcified and then implanted in a paraffin block. Decalcification was performed using 10% EDTA (ethylenediaminetetraacetic acid) at room temperature for 14 days, with the solution changed every 2 days to guarantee full decalcification. Hematoxylin and eosin (H&E) staining was applied to the sections following deparaffinization and rehydration with graded alcohol. The stained sections were scanned with a KF-PRO-005-EX scanner (KFBIO, China) and assessed using the KViewer software (KFBIO, version 1.3.1). Two independent researchers examined the histology pictures to guarantee repeatability and consistency.

### Serum analysis

Serum alkaline phosphatase (ALP), tartrate-resistant acid phosphatase (TRAP), and type Ⅰ collagen carboxy-terminal peptide (S-CTX) levels were assessed using an enzyme-linked immunosorbent test (ELISA) as per manufacturer’s instructions (ml-bio, Shanghai, China). To ensure repeatability, all experiments were done in duplicate, and absorbance was measured at 450 nm with a microplate reader (Bio-Rad, USA, model 680). Standard curves were constructed for each biomarker by serially diluting the manufacturer’s standard.

### Microbial sample collection and DNA extraction

To avoid contamination, microbial samples were gathered with painstaking care under sterile circumstances. Oral swabs were collected by gently rubbing sterile cotton swabs across the rats’ buccal and gums for approximately 30 seconds. Fecal samples were obtained straight from the rats’ rectums while they were alive and under low stress settings. Both types of samples were immediately transferred to sterile tubes and preserved in liquid nitrogen (-196°C).

DNA was extracted using a commercial extraction kit (Qiagen DNA Stool Mini Kit, Qiagen, Germany) according to the manufacturer’s instructions. Briefly, samples were thawed on ice before being mechanically disturbed to lyse the microbial cells using a bead-beating method. The lysate was treated to eliminate proteins and other impurities, enabling for the extraction of high-quality microbial DNA. The concentration and purity of extracted DNA were determined with a NanoDrop spectrophotometer (Thermo Fisher Scientific, USA) and confirmed by 1% agarose gel electrophoresis. The isolated DNA was then kept at -20°C until PCR amplification and sequencing.

### PCR amplification and sequencing

16S rRNA genes of distinct regions were amplified used specific primer with the barcode. All PCR reactions were carried out with TransStart® FastPfu DNA Polymerase (TransGen Biotech). Begin by mixing equal volumes of a 1X loading buffer, which contains SYBR Green, with the PCR products. Conduct electrophoresis on a 2% agarose gel to detect the products. The PCR products are then combined in equal density ratios. Purify the mixed PCR products using the QIAquick® Gel Extraction Kit from QIAGEN. The sequencing libraries are prepared using the SMRTbell™ Template Prep Kit (PacBio), following the manufacturer’s guidelines. Assess the quality of the library using the Qubit® 2.0 Fluorometer (Thermo Scientific) and the FEMTO Pulse system. Finally, sequence the library on the PacBio Sequel platform.

### 16S rRNA gene sequencing analysis

The 16S rRNA gene Raw sequences were initially processed through the PacBio SMRT portal (version 10.1). Sequences were filtered (minimum full pass =  3, minimum predicted accuracy =  0.9). A predicted accuracy of 90% was used as the threshold, below which a Circular Consensus Sequence (CCS) read was considered noise. The files generated by the PacBio platform were then used for amplicon size trimming, removing sequences outside the expected amplicon size range (minimum length =  1340 bp, maximum length =  1640 bp). Reads were assigned to samples based on their unique barcodes and truncated by removing the barcode and primer sequences. All representative reads were assigned to taxa using the Ribosomal Database Project (RDP) classifier (confidence threshold =  70%) against the Silva database (version 138.1).

The reads were compared with the reference database using UCHIME algorithm (UCHIME Algorithm,http://www.drive5.com/usearch/manual/uchime_algo.html) [21] to detect chimera sequences, and then the chimera sequences were removed [22]. Then the Clean Reads finally obtained.

### Statistical analysis

The statistical analyses were performed using GraphPad Prism 9 (GraphPad Software, San Diego, CA, USA). To assess differences between two groups, we applied either a two-tailed Student’s t-test for parametric data or the Mann–Whitney U test for nonparametric data. For comparisons involving more than two groups, a one-way ANOVA was utilized for parametric data, whereas the Kruskal–Wallis test was used for nonparametric data, followed by Bonferroni’s post hoc test for multiple comparisons. Statistical significance was set at p <  0.05.

Sequences analysis were performed by U parse software (U parse v7.0.1001, http://drive5.com/uparse/) [23]. Sequences with ≥ 97% similarity were assigned to the same OTUs. Representative sequence for each OTU was screened for further annotation. For each representative sequence, the SSUrRNA Database [24] of Silva Database (https://www.arbsilva.de/) [25] was used based on Mothur algorithm to annotate taxonomic information. In order to study phylogenetic relationship of different OTUs, and the difference of the dominant species in different samples(groups), multiple sequence alignment were conducted using the MUSCLE software (Version 3.8.31, http://www.drive5.com/muscle/) [26]. OTUs abundance information were normalized using a standard of sequence number corresponding to the sample with the most sequences. Subsequent analysis of alpha diversity and beta diversity were all performed basing on this output normalized data.

Alpha diversity, reflecting species diversity within a sample, was quantified using indices such as Observed-species, Chao1, Shannon, and Simpson, calculated via QIIME (Version 1.9.1) and visualized in R software (Version 2.15.3).

Beta diversity analysis, assessing differences in species complexity across samples, was performed using weighted and unweighted UniFrac metrics calculated by QIIME software (Version 1.9.1). Principal Coordinate Analysis (PCoA) was conducted to extract and visualize principal coordinates from multidimensional data. A distance matrix of weighted or unweighted UniFrac among samples was transformed into a new set of orthogonal axes, where the primary axis represented the greatest variation. PCoA results were displayed using WGCNA, stat, and ggplot2 packages in R software (Version 2.15.3).

This study employed LEfSe (Linear Discriminant Analysis Effect Size) and heat-map analysis. Sample feature data were collected and formatted for LEfSe to identify features with significant differences between categories, along with their LDA scores. Subsequently, heat-map analysis was performed on standardized data using R software (Version 2.15.3), visualizing data intensities and clustering relationships among samples and features.

## Results

### OVX reduces bone density of femur

**Fig 1:**
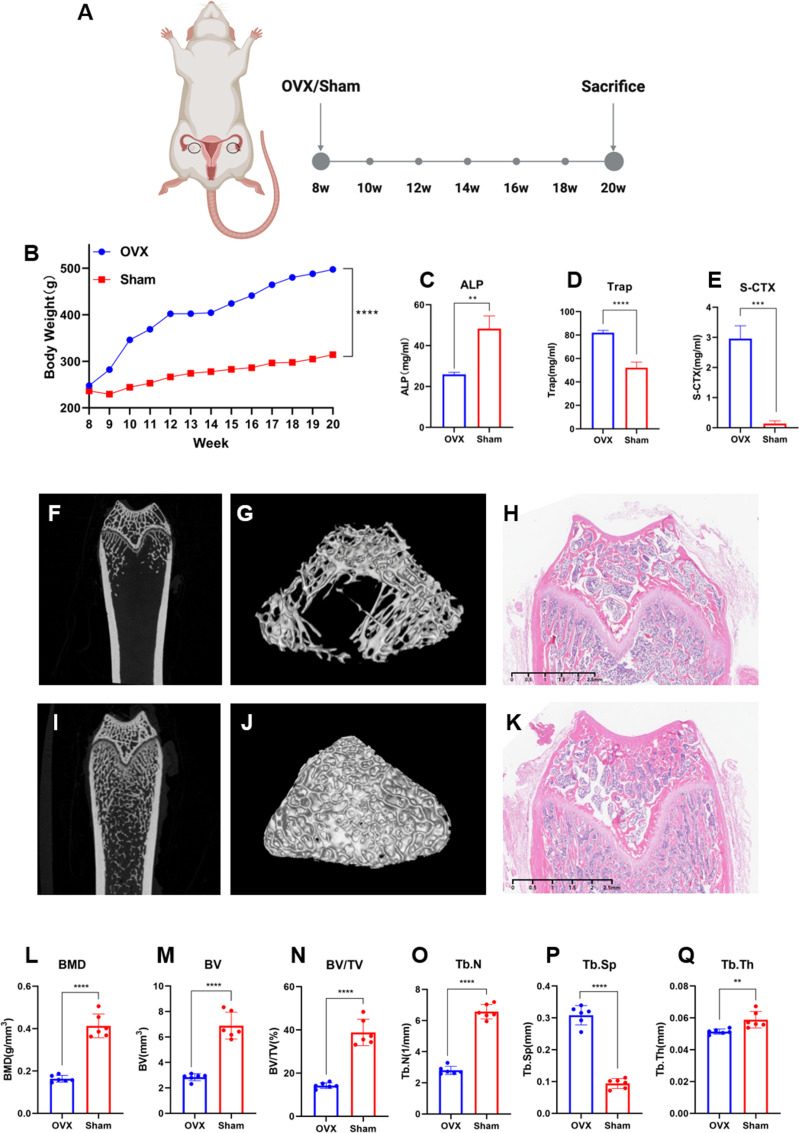
OVX educes bone density of femur. (A) Schematic diagram for the experiment. (B) Rat body weight change plotted as a line chart. (C) ALP, (D) TRAP, and (E) S-CTX levels in serum of OVX and Sham rats. Representative reconstruction of trabeculae in the (F), (G) OVX and (L), (M) Sham rat femurs used for analysis. (H) and (K) Representative pictures of H&E-stained femurs. Scale bar=500 mm. (L-Q) Quantitative analysis of bone-related parameters. Compared to Sham rats, OVX tats had a significant decrease in bone mass and impaired bone micro structure: (L) BMD, (M) BV, (N) BV/TV, (O) Tb.N,(P) Tb.Sp, and (Q) Tb.Th. Data are presented as the mean ± SD. Tb trabecular bone. * *p* < 0.05, ** *p* < 0.01, *** *p* < 0.001, and **** *p* < 0.0001.

Fig 1A shows a diagram of the experimental design. Compared to the Sham group, the body weight of OVX rats increased rapidly following OVX surgery (*p* <  0.001) (Fig 1B). OVX rats had significantly higher levels of tartrate-resistant acid phosphatase (TRAP; *p* <  0.0001) (Fig 1C), type I collagen carboxy-terminal peptide (S-CTX; *p* <  0.001) (Fig 1D), and significantly lower levels of alkaline phosphatase (ALP; *p* <  0.01) (Fig 1E).Micro-CT examination revealed considerable degradation in the femoral trabecular bone structure of Sham rats (Fig 1F–H) as compared to OVX animals (Fig 1I–K). OVX rats had significantly lower bone mineral density (BMD; *p* <  0.0001), bone volume (BV; *p* <  0.0001), and bone volume-to-total volume ratio (BV/TV; *p* <  0.0001) (Fig 1L–N). Trabecular number (Tb.N) fell dramatically (*p* <  0.0001) (Fig 1O), whereas trabecular spacing (Tb.Sp) increased (*p* <  0.0001) (Fig 1P) and trabecular thickness (Tb.Th) decreased (*p* <  0.01) (Fig 1Q). Histological examination with hematoxylin and eosin (H&E) staining revealed sparse and fragmented trabeculae, as well as lower bone density in OVX rats compared to Sham animals.

### OVX changes the diversity of oral microbiota

**Fig 2:**
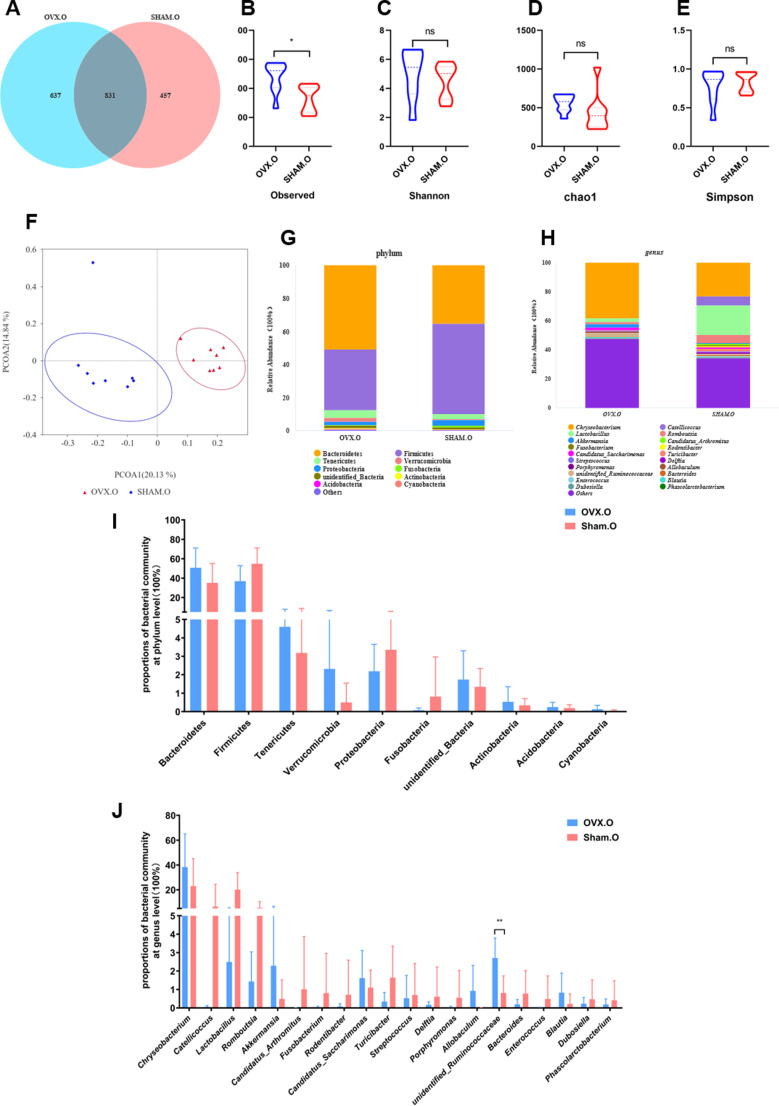
OVX changes the diversity of oral microbiota. (A) Venn diagram, (B) observed index, (C) Shannon index, (D) Chao1 index, and (E) Simpson index in OVX and Sham rats. (F) PCoA of the two groups of rats. Differences in the oral microbiota between the OVX and Sham rats at the phylum level (G and I) and the genus level (H and J). * 0.01 < *p* ≤ 0.05; ** 0.001 < *p*≤0.01; *** 0.0001 < *p* ≤ 0.001, **** *p* < 0.0001.

The oral microbiome changed following OVX surgery. A Venn diagram (Fig 2A) depicted the shared and unique OTUs between the OVX and Sham groups. The results revealed that both group’s oral microbiotas shared 831 OTUs, with the Sham group comprising 457 unique OTUs and the OVX group including 637 unique OTUs. Alpha-diversity (α-diversity) indices were used to evaluate microbiological diversity within groups. The OVX group had significantly increased diversity, as indicated by the observed species index (*p* <  0.01) (Fig 2B), Chao1 index (*p* <  0.05) (Fig 2C), and Shannon index (*p* <  0.001) (Fig 2D). However, there was no discernible variation in the Simpson reciprocal index between the two groups (Fig 2E). These data point to an increase in microbial richness and evenness in OVX rats. Principal coordinate analysis (PCoA) showed unique clustering of oral microbiota composition between the OVX and Sham groups, indicating significant species composition variations (Fig 2F). At the phylum level, Bacteroidetes and Firmicutes made up more than 90% of the total oral microbiota in both groups. Following OVX surgery, Bacteroidetes rose dramatically, while Firmicutes decreased (*p* <  0.05) (Fig 2G,I). At the genus level, OVX rats had a significantly higher relative abundance of *Chryseobacteriu*m (*p* <  0.01) and *unidentifined Ruminococcaceae* (*p* <  0.01) than Sham rats. In contrast, the OVX group had considerably lower levels of *Lactobacillus* (*p* <  0.01) and *Romboutsiawas* (*p* <  0.05) (Fig 2H,J).

### Significance analysis of gut bacterial community abundance after OVX

**Fig 3:**
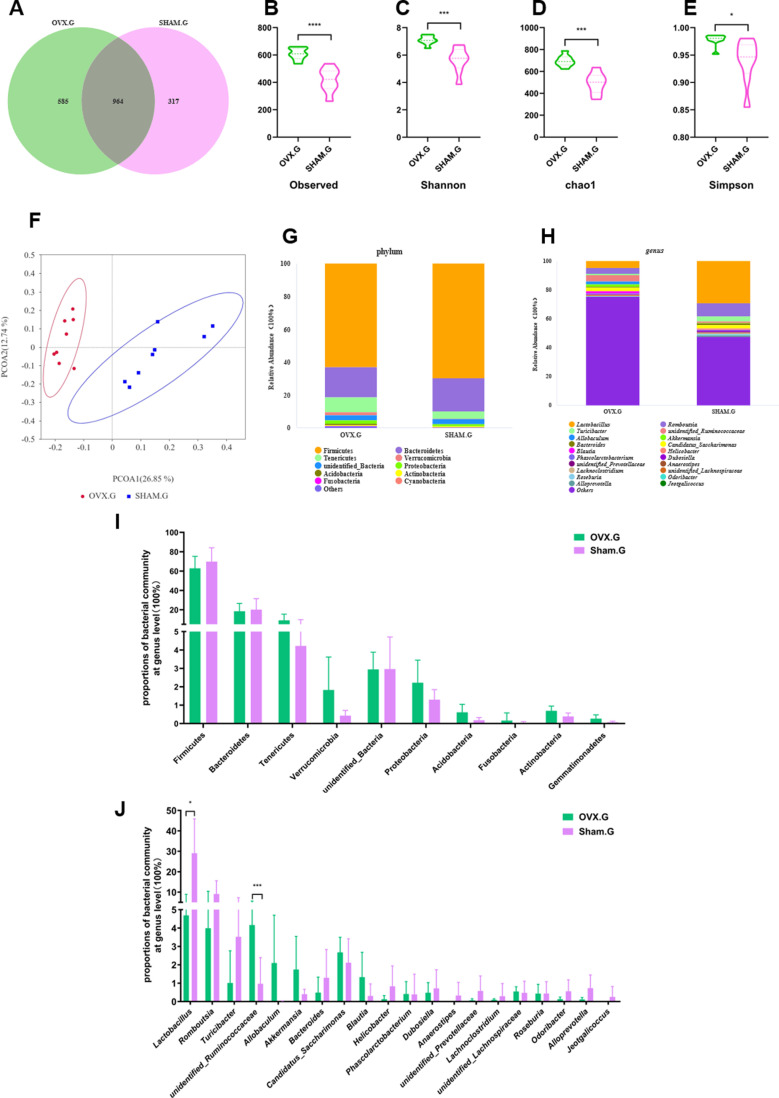
OVX changes the diversity of gut microbiota. (A) Venn diagram, (B) observed index, (C) Shannon index, (D) Chao1 index, and (E) Simpson index in OVX and Sham rats. (F) PCoA of the two groups of rats. Differences in the gut microbiota between the OVX and Sham rats at the phylum level (G and I) and the genus level (H and J). *0.01 < *p* ≤  0.05;**, 0.001 < *p* ≤ 0.01;***, 0.0001 < *p ≤* 0.001;****, *p* < 0.0001.

The gut microbiota of the OVX and Sham groups shared 964 OTUs, with the OVX having 585 unique OTUs and the Sham group having 317 unique OTUs (Fig 3A). Alpha-diversity indices revealed that the OVX group had much greater microbial variety than the Sham group. OVX rats showed significantly greater species index (*p* <  0.0001) (Fig 3B), Shannon index (*p* <  0.001) (Fig 3C), Chao1 index (*p* <  0.001) (Fig 3D), and Simpson index (*p* <  0.05) (Fig 3E). The principal coordinate analysis (PCoA) (Fig 3F) revealed comparable small within-group distances for the OVX and Sham groups, indicating similar species compositions within each group. However, the clear divergence between the two groups indicated significant variations in species composition between OVX and Sham rats. Bacteroidetes and Firmicutes were the most common phyla in the gut microbiota, accounting for more than 80% of the total microbial population in each category. Significant differences were found at both the phylum and genus level. The OVX group had higher relative abundances of Acidobacteria (*p* <  0.05), Actinobacteria (*p* <  0.05), and Gemmatimonadetes (*p* <  0.05) over the Sham group. OVX rats had considerably larger abundances of *unidentifined Ruminococcaceae* (*p* <  0.001), but lower abundances of *Anaerostipes* (*p* <  0.05) and *Alloprevotella* (*p* <  0.05) compared to Sham rats.

### Research on the correlation between oral and gut microbiota

**Fig 4:**
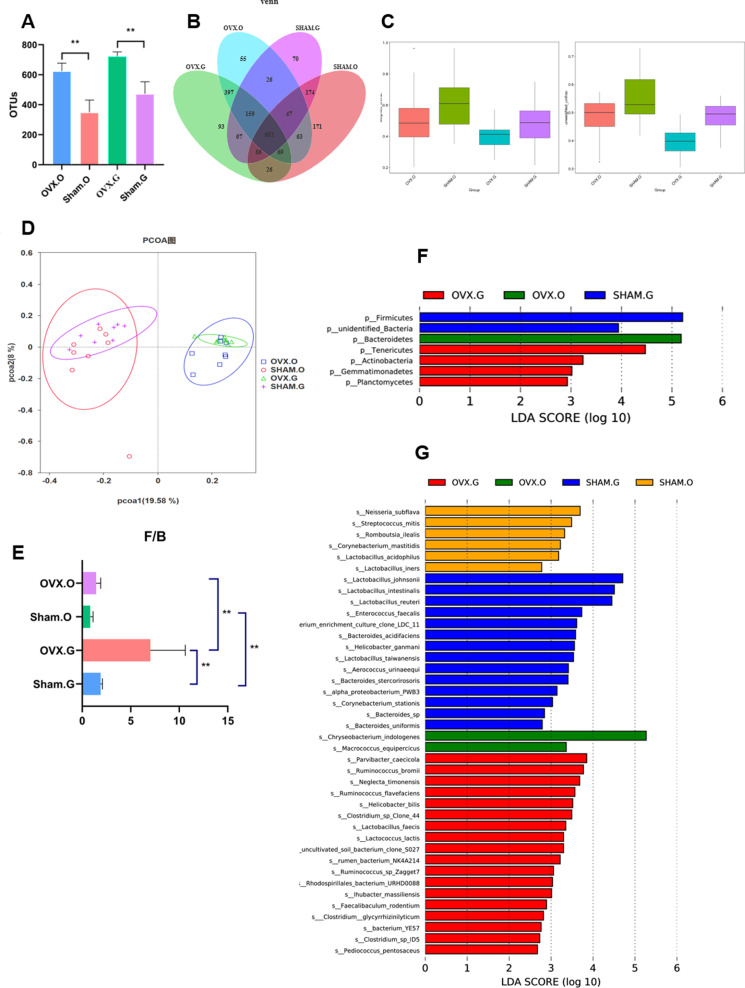
Correlation between oral and gut microbiota. (A) OTUs of oral and gut microbiota in OVX group and Sham group, (B) Venn diagram shows the difference in OTUs of four groups, (C) α-diversity of four groups, (D) PCoA of four groups, (E) F/B ratio, (F) LEfSe bar at genus level. (G) LEfSe bar at phylum level, LDA value > 4. ^*^0.01<*p*≤0.05, ^**^0.001<*p*≤0.01, ^***^0.0001<*p*≤0.001, ^****^p<0.0001.

According to sequencing data, the oral and intestinal microbiota in the OVX group were classified into 571 and 698 OTUs, respectively, whereas the Sham group was classified into 385 and 505 OTUs (Fig 4A). The number of bacterial taxa was inversely linked with BMD (*p* <  0.01). A Venn diagram (Fig 4B) demonstrated that 1,277 OTUs were shared by the oral and gut microbiotas in OVX rats, with 55 OTUs unique to the oral microbiota and 93 OTUs unique to the gut microbiota. In comparison, the Sham group had 959 common OTUs, including 171 unique to the oral microbiota and 70 unique to the gut microbiota. The β-diversity (Fig 4C) study revealed a significant decrease in microbial composition heterogeneity among samples in the OVX group. The PCoA analysis revealed substantial within-group similarity in both the OVX and Sham groups, but strong clustering between the two was detected, indicating significant variations in microbial composition. The Firmicutes/Bacteroidetes (F/B) ratio, a key measure of gut microbial health, increased considerably following OVX (*p* < 0.01) (Fig 4F). Both groups had a higher F/B ratio in the stomach than in the oral cavity (*p* <  0.01 for both). Significant changes in microbial composition across the groups were identified by LDA and LEfSe analyses at the taxonomic level, as well as through effect size measurement. The oral microbiota of OVX rats was enriched with 1 phyla and 2 genera, whereas the gut microbiota was enriched with 4 phyla and 18 genera. In the Sham group, no phyla or 6 genera were enriched in the oral microbiota, whereas the gut microbiota was enriched by 2 phyla and 14 genera.

**Fig 5:**
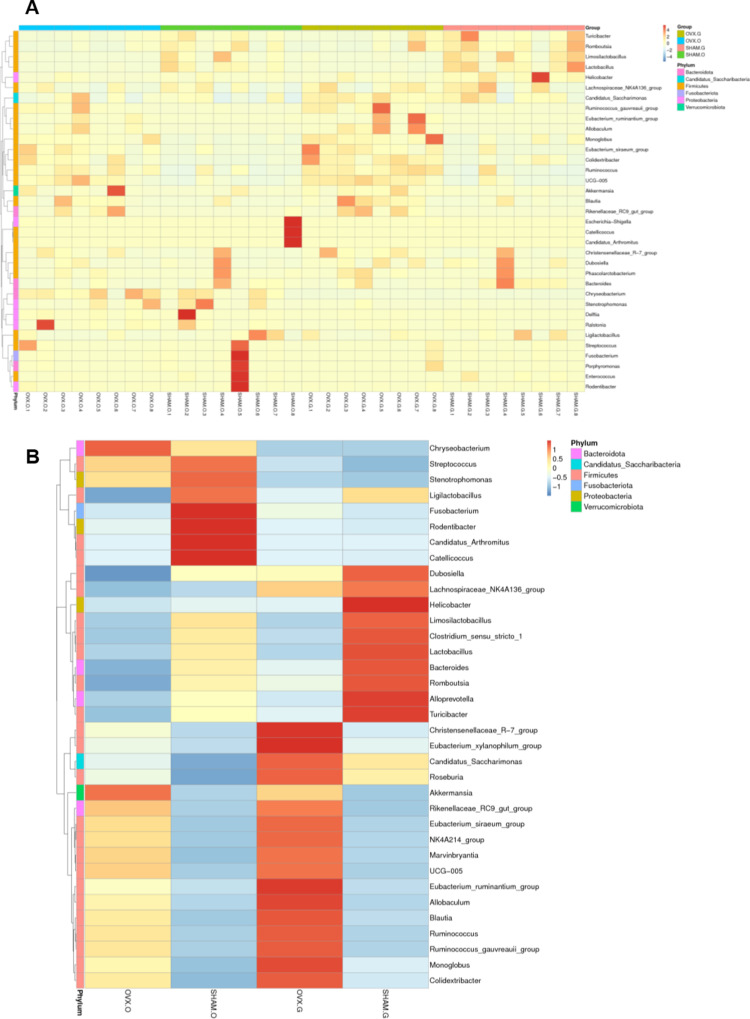
The heatmap analysis of the oral and gut microbiota. (A) Heatmap showing the top 35 differential of microbiotas of all species. (B) Heatmap showing the top 35 differential microbiotas of four groups.

The heat-map analysis of the oral and gut microbiota (Figs 5A,B) revealed substantial variations in microbial composition between the OVX and Sham groups. Specific bacterial taxa were far more prevalent in the OVX group than in the Sham group.

## Discussion

Osteoporosis (OP), a prevalent metabolic bone condition characterized by low bone mass and structural degeneration, is predominantly caused by estrogen insufficiency after menopause, which affects bone turnover by increasing resorption and decreasing creation [27–29]. Beyond its skeletal effects, OP has been connected to changes in the oral and gut microbiota, both of which play important roles in metabolism, immunological regulation, and systemic inflammation—all of which influence bone health [12,30–32 ]. The oral-gut microbiota axis, which is defined by bidirectional interactions between oral and gut microbial communities, has been linked to systemic bone metabolism and the development of bone-related disorders [33–35]. To investigate these associations, this work used the bilateral OVX model, a proven approach for imitating postmenopausal estrogen insufficiency and OP in animal studies [36]. The OVX model’s ability to reproduce hormonal and metabolic alterations, as well as its impact on the microbiota, makes it ideal for studying the function of the oral-gut microbiota axis in OP pathogenesis and its possible consequences on systemic bone metabolism.

### Impact of OVX on bone microstructure and serum markers

OVX caused considerable bone loss and microstructural degeneration in rats, as revealed by micro-CT analysis, consistent with prior research [37,38]. Reductions in bone mineral density (BMD), bone volume (BV), and bone volume-to-total volume ratio (BV/TV) found in OVX rats are consistent with postmenopausal osteoporosis (OP) characteristics. In addition, trabecular number (Tb.N) and thickness (Tb.Th) dropped, whereas trabecular spacing rose. These structural changes were accompanied by considerable changes in serum indicators of bone metabolism. In OVX rats, elevated levels of tartrate-resistant acid phosphatase (TRAP) and type I collagen carboxy-terminal peptide (S-CTX) indicate increased bone resorption activity, but low levels of alkaline phosphatase (ALP) indicate decreased bone production. Estrogen has an important role in bone health by inhibiting osteoclast activity and increasing osteoblast function. However, the reduction in estrogen levels after menopause upsets this equilibrium, resulting in increased bone resorption (high TRAP and S-CTX levels) and decreased bone production (lower ALP levels). These metabolic alterations play an important role in the development of osteoporosis [39]. The study’s findings are consistent with previous research, confirming the OVX model as a credible and reproducible description of postmenopausal osteoporosis [36].

### Serum markers and microbiota interactions

Significant variations in blood indicators of bone metabolism seen in OVX mice could be attributed to microbiota abnormalities. The enrichment of pro-inflammatory taxa, combined with a decrease in helpful bacteria such as Lactobacillus, most likely correlates to elevated TRAP and S-CTX levels, indicating accelerated bone resorption. Conversely, a decrease in ALP levels corresponds with decreased bone formation, which may be aggravated by microbial dysbiosis [20,36].

The gut microbiota’s influence on bone health is further highlighted by its function in inflammatory pathways and immune modulation. The bone remodeling process may be disrupted by dysbiosis-induced systemic inflammation, which links microbial shifts to changes in serum markers and bone loss in OVX rats [40,41].

### Oral-gut microbiota axis and bone health

The human body is home to trillions of microorganisms, with the gut microbiota being referred to as a “key organ” because of its vital function [42]. In order to maintain general homeostasis and immunological function, the gut microbiota controls metabolic phenotypes, epithelial cell growth, and innate immunity [43]. Through immune modulation, it also has a major effect on bone metabolism [40], which effects bone health and OP pathology [41,44,45]. Primary OP is linked to the enrichment of particular bacteria, such as Bacillus and Faecalibacterium, which increase bone mass by suppressing osteoclast activity and fostering osteoblast function [46,47]. The second biggest microbial community, the oral microbiome, has a significant impact on health as well [48,49]. Systemic inflammation brought on by oral microbiome dysbiosis, especially when it involves pathogens like Porphyromonas gingivalis, can result in bone loss and an elevated risk of fracture [35].

Changes in the oral microbiome can impact the gut microbiota, which in turn can impact systemic bone metabolism, as the oralgut microbiome axis emphasizes [33,34]. The observed changes in microbiota composition are consistent with earlier research showing that gut dysbiosis can worsen oral dysbiosis, which is frequently characterized by an increase in periodontal infections. This can intensify systemic inflammation and metabolic disturbances linked to bone loss. Additionally, chemicals produced by gut microbes are essential for preserving bone health and affecting the balance of oral microbes. The pathophysiology of conditions including diabetes, rheumatoid arthritis, and colorectal cancer is influenced by this interaction [50–52].

Ovariectomy (OVX) caused considerable changes in the variety and composition of both the oral and intestinal microbiota [53]. Alpha-diversity indices, such as Shannon and Chao1, were considerably higher in OVX rats, indicating greater microbial richness and evenness [54], albeit this does not always imply a healthy microbiome, as enhanced diversity can often reflect dysbiosis in sick situations. The Firmicutes-to-Bacteroidetes (F/B) ratio, a key microbial balance marker, was significantly elevated in OVX rats, particularly in the gut, a shift that has been linked to systemic inflammation and metabolic disorders, which may exacerbate bone resorption and contribute to osteoporosis progression [45,55,56]. A considerable decline in Lactobacillus, a probiotic crucial for maintaining microbial balance, suppressing oral infections, lowering peri-implant inflammation, and maintaining gut barrier integrity, was also noted [41,44]. *Lactobacillus* deficiency is likely to worsen systemic inflammation and jeopardize bone health because it is essential for regulating inflammatory responses and promoting bone metabolism. Concurrently, the abundance of Bacteroidetes, a phylum commonly associated with inflammatory disorders, in both oral and gut microbiota underlines its possible role in generating systemic inflammation in estrogen-deficient situations. These findings highlight the complex relationship between estrogen deficiency, microbial dysbiosis, and bone health, with observed microbial shifts-particularly the loss of beneficial taxa like Lactobacillus and the proliferation of pro-inflammatory microbes like Bacteroidetes-implying that dysbiosis within the oral-gut microbiota axis significantly contributes to osteoporosis pathogenesis via systemic inflammatory and metabolic disruptions.

### Phylum and genus-level alterations in microbial communities

OVX and Sham rats have significantly different microbial compositions at the phylum and genus levels. At the phylum level, Bacteroidetes, which are frequently associated with inflammatory disorders such as periodontitis and Alzheimer’s disease, rose in abundance, while Firmicutes, which are prevalent in periodontal disease, also showed significant alterations [57,58].

The OVX group exhibited an abundance of pro-inflammatory taxa, including *unidentifined Ruminococcaceae*, which stimulates osteoclast activity by releasing pro-inflammatory cytokines like TNF-α and IL-6 [59,60]. Concurrently, there was a decrease in beneficial taxa like *Lactobacillus* and *Alloprevotella*, both of which are essential for bone health. *Lactobacillus*, a well-known probiotic, has been demonstrated to decrease osteoclastogenesis and increase osteoblast differentiation by producing metabolites such as short-chain fatty acids (SCFAs), which are protective in bone metabolism [61–63]. The loss in these helpful microorganisms may impair the host’s ability to prevent bone resorption and maintain bone homeostasis.

The LDA analysis found significant microbial biomarkers that distinguished OVX from Sham rats, highlighting the impact of estrogen deprivation on microbial composition [33,34]. These findings not only highlight the significant impact of altered microbiota on bone metabolism, but also lend support to the hypothesis that microbial dysbiosis contributes to systemic bone loss by exacerbating inflammation, impairing nutrient absorption, and disrupting metabolic pathways critical to bone health [55]. This highlights the efficacy of microbiome-targeted therapies, such as probiotics or dietary changes, in preventing osteoporosis and its consequences.

### Comparison with previous studies and implications for future research

Our findings are consistent with earlier research that found increased microbial diversity and alterations in important bacterial taxa following OVX. Research on the association between α-diversity and bone health yields conflicting results, highlighting the need for more inquiry. Wang et al. showed similar increases in oral microbial diversity after OVX, although other studies found no significant changes in older persons with dental caries [54,64–66]. These disparities could be due to differences in experimental models, age, or environmental factors.

The observed changes in the F/B ratio and Lactobacillus abundance are congruent with data from human research on OP and periodontal disease, highlighting the discoveries’ translational potential. Future research should focus on understanding the mechanisms behind microbiota-mediated bone metabolism and developing treatment options that target the oral-gut microbiota axis. Probiotic therapies, particularly those involving Lactobacillus strains, may show promise for reducing bone loss and increasing overall health in postmenopausal women [44,46].

## Conclusion

This study focuses on the complex relationship between estrogen shortage, microbial dysbiosis, and bone health, with significant changes identified in both oral and gut microbiota following OVX. The increased alpha-diversity and higher Firmicutes-to-Bacteroidetes ratio in OVX rats, combined with a decrease in beneficial taxa such as *Lactobacillus*, highlight the importance of the oral-gut microbiota axis in systemic bone metabolism. These microbial alterations are associated with increased systemic inflammation and metabolic disturbances, which contribute to faster bone resorption and poor bone formation. The concentration of pro-inflammatory taxa, such as Bacteroidetes and *unidentifined Ruminococcaceae*, emphasizes the inflammatory environment created by microbial dysbiosis under estrogen-deficient circumstances. Consistent with previous studies, these findings corroborate the OVX model’s efficacy in modeling postmenopausal osteoporosis and highlight the potential of microbiome-targeted therapies, such as probiotics and dietary adjustments, in reducing bone loss and improving skeletal health. Future research should look into the molecular pathways that relate microbial dysbiosis to bone metabolism, as well as the therapeutic efficacy of altering the oral-gut microbiota axis in osteoporosis care.

## Supporting information

S1 Data OVX.G.data. (ZIP)

S2 Data OVX.O.data. (ZIP)

S3 Data SHAM.G.data. (ZIP)

S4 Data SHAM.O.data. (ZIP)
